# Landscape analysis of healthcare policy: the instrumental role of governance in HIV/AIDS services integration framework

**DOI:** 10.11604/pamj.2020.36.27.22795

**Published:** 2020-05-21

**Authors:** Maureen Atieno Adoyo

**Affiliations:** 1 Faculty of Health Sciences, Rongo University, Migori, Kenya

**Keywords:** Governance, healthcare, integration, policy, framework, Kenya

## Abstract

**Introduction:**

Low and middle-income countries HIV/AIDS interventions are yet to achieve the desired levels of health outcome due to lack of effectiveness and efficiency in programming, a challenge associated with resource limitations, fragmented services, complexities in population and disease characteristics including political landscape. The objective of this study was to establish the instrumental role of governance in the implementation of HIV/AIDS services integration policy framework, with focus on organization structure, participation in decision making, collaboration, stakeholder engagement, political commitment as study variables.

**Methods:**

Using a mixed method design, a total number of 30 health workers, 5 county AIDS services coordinators (CASCOs), 8 sub-CASCOs and 3 representatives of inter coordinating committee were interviewed in compliance with ethical protocols. Multi-stage sampling techniques was used to select counties in Kenya, health institutions and respondents. Quantitative and qualitative data was generated by administering semi structured questionnaire and key informant interview guide.

**Results:**

Generated from excel sheet and NVivo software indicate that organization structures existed and clarity and ease of work varied across the different levels of care. Collaboration efforts, however varied, created synergy in policy framework implementation and political commitment complemented the various leadership actions for successful implementation of integration policy framework.

**Conclusion:**

Governance role is indispensable in the implementation of health policy framework. Policy makers need accurate epidemiological and demographic information to implement contextualized policy framework necessary for sustained improvement in health outcomes.

## Introduction

Low and middle-income countries (LMIC) are consistently low in achievement of health outcomes measured by reduction in morbidities and mortalities; responsiveness in healthcare services; risk protection, both social and financial and efficiency in the delivery of healthcare services. The challenges are further compounded by contextual factors such as demographic dynamics in the population, epidemiological and political landscape [1]. In the past four decades, HIV/AIDS has remained a global concern as its ravaging impact continues to be felt among the afflicted population and governments. In response to the pandemic the has been rapid increase in global partnership with initial stages witnessing vertical and fragmented service delivery in which, for example VCT were stand-alone facility with its own data gathering systems. The existence of separate infrastructures arrangement had major limitations associated huge capital investment with little achievements, Loss-To-Follow-Up (LTFU) in the continuum of care, coupled with missed opportunities in counselling and testing [1,2].

To Improve efficiency, World Health Organization (WHO) and other development partners embarked on swift move to urgently reverse the trend through a scaled approach to provision of antiretroviral therapy. The approach required systematic links across primary, secondary and tertiary healthcare facilities including strengthening home-based care within the countries. Healthcare facilities had a task to deliver ART alongside others services such as, HIV prevention programmes, counseling and testing, prevention of mother to child transmission (PMTCT), tuberculosis (TB), family planning (FP) and any other health services that were essential to optimal antiretroviral treatment [3]. Countries have since responded to integration calls and have adopted various implementation strategies and guideline. For example, Kenya AIDS strategic framework (KASF) 2014-2018 outlines the need to maximize efficiency in service delivery through integration and creation of synergy among HIV prevention and treatment programmes.

The basic feature of an integrated and effectively operating healthcare institution is one with the ability to maximize the delivery of a range of medical and preventive interventions such as counseling and testing, PTMCT, MCH, FP, ART, Control of TB and STI services, this therefore means, services are packaged and delivered at a single point or co-located alongside other health care services [4]. Principle underpinning integration framework for delivery of quality services to all people, where and when they need, puts emphasis on coordination within health systems, to ensure uninterrupted service delivery. For health managers, this meant increasing access by putting in place strategies that reduce physical distance, financial and administrative barriers, this also required deliberate efforts in community engagement in delivery of treatment and support services [5-7]. Literature review, show there is no universal integration framework agreed upon but model success under implementation depended on understanding of contextual factors in utilization of services across the continuum of care [7].

**Figure 1:**
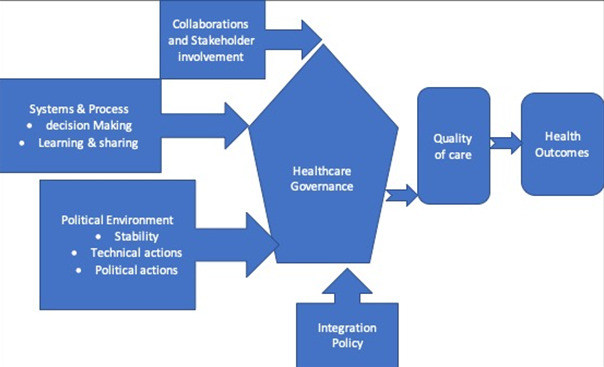
Healthcare governance framework

In the same context, countries vary enormously in terms of geography, epidemics, politics, economics and culture that determines the organization of their health systems. Additionally, a country´s health policy framework mirrored social pressures, as well as national values and priorities depending on disease prevalence [8]. To contextualize the study, Figure 1 Illustrates healthcare governance framework and the interrelationship between the various components. Governance, a guiding principle for integrated services elicits high expectations upon leadership to take technical and political actions to ensure strategic policies exist, formation of collaborations and coalition in systems designs including leveraging resources to reconcile competing demands of limited resources. In order to achieve sustained effort in health systems strengthening, WHO emphasizes on good governance and stewardship as opposed to traditional “command and control" approach among leadership. Good governance, allows public space for active participation and rightly demand for greater say in the management of health services needed.

The result is greater access to services that is appreciated by clients and services provider and so achieving “quality of care we want" becomes a reality and not a dream [1,9,10]. This study explored healthcare governance by four dimensions namely, organizational structure; participation in decision making; collaboration; stakeholder engagement; political commitment and action. Organizational structures are drivers to the implementation of strategies including policies. They define rules, roles and responsibilities that makes it possible to determine feasible and optimal action to be undertaken. Healthcare systems requires high-level coordination of workflow to allow different stakeholders; community, health workers, donors and politicians to harmoniously work together. The workflow describes the hierarchical order and reporting lines and a well-designed structure eliminates potential contradictions likely to arises from confusion in roles, failure to share ideas and conflict arising from complexities in decision making process [11,12].

Collaboration between implementing agencies in HIV/AIDs services arena has seen partners use standardized and unified HIV/AIDS data collection, with specified common key indicators to measure implementation and effectiveness of care. Additional effort has put to assemble, review and revise variables against the key indicators developed in the national monitoring and evaluation system [13]. The missing link here, is the documentary evidence showing collaboration and information sharing among partners implementing HIV/AIDs programme has had impact on integration aimed at improving access and efficiency of HIV/AIDs care. Power for decision-making focuses on acts of individuals and groups in influencing policy decisions. Classical studies indicated variance in the way power is conferred to citizens and interest groups were unequally distributed [10]. Stakeholder are at different levels in decision making and their opinion shape up policy process. Thus, power relation is deemed critical in the determination of healthcare systems organization.

Generally, players both at local and international levels look upon the government to take leadership in policy development, championing, partner coordination and implementation of health policies. Political commitment is reflected in agenda setting and action by the governments or institutional leadership. The emerging trend from the literature reviewed, demonstrates that attainment of public health goals is dependent on political events which vary in time and region [14]. This therefore underpins the importance of making comparison in governance among the different institutions providing HIV/AIDS care services. The comparative information would then demonstrate how different institutional arrangement and leadership are critical in the implementation of health policy and quality of services delivered. To this extent, this study aimed at assessing how these governance dimensions play an instrumental role in the implementation of HIV/AIDS services integration framework.

## Methods

### Study design

this study was predominantly a mixed method design, utilizing both quantitative and qualitative approach in data collection and analysis. The design was important in providing an account for differentials in the implementation of HIV/AIDS services Integration framework in different counties and levels of care, including description of institutional culture and individual characteristics of healthcare leadership, health workers, donor agencies and the community [15]. The choice of design was based on the strength to produce knowledge base, which would act as foundation for more rigorous analytical study, applying experimental manipulation to quantify variations in integration implementation status as result of measurable governance actions. A total of 30 health works (HWs), 5 county AIDS services coordinators (CASCOs), 8 sub- CASCOs and 3 representatives of inter-agency coordinating committee were interviewed between June and October 2017.

### Setting

the respondents were spread out across five counties of Kenya, purposively selected based on the unique population seeking for HIV/AIDS services. The selection characteristic included counties with majority numbers of key populations, minorities with tightly neat culture, high prevalence county and a cosmopolitan city. Health facilities were stratified as referral and health centers to give richness in the different level of health care services. From each strata the facilities/hospitals were randomly selected for inclusion in the study. Table 1 shows study counties and Health Facilities. The health workers working in comprehensive care centers who were on duties during the interview days were all included in the study.

**Table 1:** List of facilities selected for the study

	County	Facility Name
1	Homabay	Nyatoto Health Center
		Ndhiwa Sub county
		Mbita Sub county
2	Nairobi	Mama Lucy sub county
		Makadara Hospital
		Kibra health center-Amref Clinic
3	Mombasa	Potriez Sub County Hospital
		Mwatapa Health Center
		Likoni District Hospital
4	Kajiado	Ngong Sub County Hospital
		Kimana Health Center
		Loitoktok Sub-County Hospital
5	Isiolo	Isiolo County Hospital
		Merti Health center
		Farfi Sub-county

### Data collection

semi structured questionnaire with three different thematic section was administered to HWs and the questions were sequenced to build from preceding section to ensure consistency checks in responses. Key informant interviews (KIIs) were conducted with hospital and comprehensive care centers (CCCs) in charges and representatives of ICCs. Both quantitative and qualitative approaches allowed for in-depth understanding of issues related to integration policy implementation, governance, challenges and opportunities in HIV/AIDS services integration.

### Data analysis

analysis of quantitative and qualitative data was done concurrently. Quantitative data from health worker questionnaire captured using open data kit (ODK) were cleaned and made available for analysis on excel sheet. The summary data, were presented as charts and graphs and mainly describing trends of an events in healthcare institution. Qualitative data from KIIs were coded both manually and using N-VIVO computer programme. The study adopted a systematic approach of reducing qualitative data, categorizing data based on study or emerging themes and patterns after analysis of specific statements. Mapping and interpretation of the texts was done to identify meaningful and relevant findings, which was reported as a blend of extract narratives or excerpts of the condensed meanings or quotes that provided evidence to support the study claims where necessary.

### Ethical consideration

since the study dwelt on a sensitive area of healthcare provision, prior approval from scientific review committee was sought and obtained. The work ethic and rights of all the institutions involved were respected and they provided a written permission before commencement of the study. Full disclosure on study purposes, benefits risks was done at institutional and individual respondent level. Participation in the study was voluntary and respondents we given opportunity to a written consenting process. Anonymity and confidentiality were observed through the entire study process as the respondents were tagged to unique identifiers.

## Results

### Organisation structure and operations

**Figure 2:**
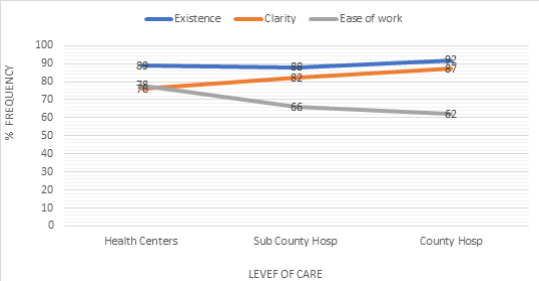
Description of organization structure

results in Figure 2 describe organization structure existence, clarity of such structure and ease of work within such structure. The response indicated consistency that organization structures do exists in the different level of service delivery ranging 89-92% at health centre to county referral level. Clarity of structure was witnessed to have an increase in ascending order from health centre to county (70-87%), while ease of work was reported to be high (70%) at health centre level and decreased systematically to sub-county level and to county level with 66% and 62% respectively.

When key informants were asked about organisation structure and leadership action at the advent of integration policy implementation, some of the respondents explained: “ …*MOH is the overall in charge, in a sub county hospital, then we have the departmental heads under the MOH, health facilities has the clinician, the laboratory in charge, we have the nursing officer in charge, we have the nutritionist, therapist and maintenance person. Then below them we also have other sub departmental in charges like now we have the CCC in charge and we also have somebody in charge of the maternity, somebody in charge of the PMTCT…”* MBT 1.

“*…Integration and say devolution has brought improvement in leadership and issues of reporting uh and how the information flows because after integration you have somebody (a point person) who is in charge for that so that if there is any problem they can report to the immediate in charge and then the in charge takes it over and may be report to the next person.”* KWG 8.

The health institutions are reported to have organisation structure with leadership taking charge of the different section as stated by a health worker. “…*at various departments, we have focal people or focal persons that should be providing leadership. Uhh or taking ownership of the implementation of the services…”* LKN 8.

Additionally, functional organization structure promoted information flow and coordination needed for smooth and efficient operation in delivery of HIV/AIDs services as explained by one of the key informants:

“*In terms of leadership in integration, we see how using the same policy documents and how different actors contributing in terms of state and non-state actors, I would give an example like for services targeting elimination of mother to child transmission PMTCT…”* ICC 3.

### Collaboration and stakeholder engagement

**Figure 3:**
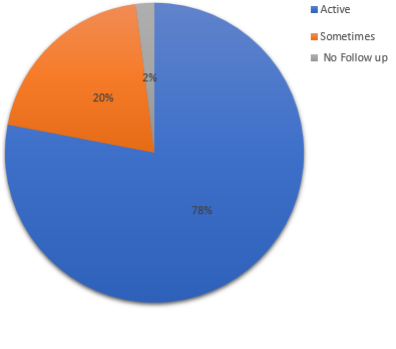
Contact or follow up efforts in collaboration

**Figure 4:**
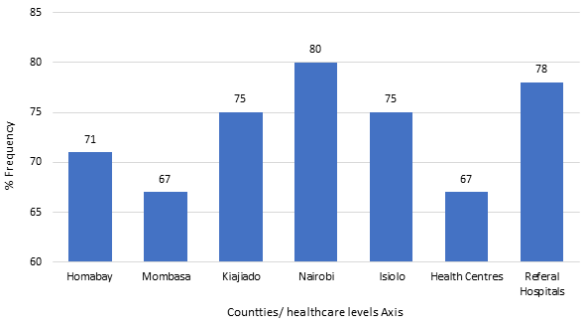
Collaborative arrangements between counties and facilities

to assess collaboration and level of stakeholder involvement in the implementation of integration policy, key informants cited efforts by county level leadership and facilities to work in partnership through engagement of stakeholder and this resulted to synergy creation, particularly in human resource and health information management. Respondent explain collaboration scenario before and during implementation of integration policy: “*…HIV care was actually seen to be a program based, that the Ministry of Health per say then did not take key role in terms of this care, so partners would come to government health facility and start their activities, but you see people working for the Ministry were really not keen in terms of ownership of the care for HIV. So, I think when you talk about partnership of the Ministry of Health and other donor agencies it has improved, in terms of health care provision, now you can see both Ministry employed and partner employed personnel are working together.” (MBT 1) “In face of integration. I think, stakeholders have really tried to support, we do not have any problem with the stakeholders everybody is supporting integration in terms of HIV care and management.”* LTK 3 To further show strength of relationship among partnering entities, the respondents were asked if there was an attempt to have regular contact or follow up activities with collaborating institutions and majority (80%) of the health institutions both health center and hospitals combined, had active/vibrate relationship (Figure 3). Further interrogation on nature of collaboration as indicated in Figure 4, revealed that referral hospitals engaged more in collaborations compared to health centers at 78% and 67%. Results analyzed by counties showed that Nairobi (capital city) had the highest level of collaboration while Mombasa registered the lowest levels of collaboration at 80% and 67% respectively. Collaboration was mainly between facilities and donor agencies or facilities to hospitals as expressed by a number of key informants: “…*partnership of the Ministry of Health and other donor agencies it has improved, in terms of health care provision…”* MBT 1 “…*we have a multi sectoral representation. So we have partners both bilateral and multilateral partners*. “ ICC 1 “…*we refer for let´s say nutrition to other clinics here in the hospital and other special care we ask them to go to county hospital*…. “ MSA 2. Collaboration arrangement was reported to be well coordinated and systems put in place seemed to be working harmoniously among the different actors involved in treatment and care, one of the respondents had this to say: “..*we implement on what has been agreed as service delivery institutions and the partners, mostly do the trainings and when there are any changes in the directions of care, then we come together and we can discuss. When there are new updates, they call all the participants institutions and share update. The community health management board are also involved, I remember when they were rolling out the strategic plan for HIV, there were religious leaders, chiefs, county leaders, community leaders, people living with disabilities* … ” ISL 3.

### Level of participation in decision making

**Figure 5:**
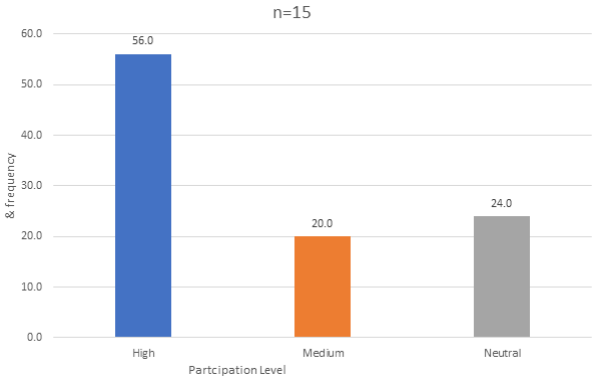
Level of staff participation in decision making process

results in Figure 5 indicated that Involvement or participation of stakeholders in decision making at various levels in operation process was cumulatively found be on averagely high at 56% compared to medium level of participation at 20% and others who did not have clear opinion were reported as neutral at 24%. The few respondents (20%), who felt their participation in decision making was at medium level gave indicative reasons of such perception, which included resources need which had not been met and their opinions not reflected in the final decision. Key informants said: “…*level of participation is not enough compared to what is needed in the current situation… to some extent they have not given us what we said we need.”* MSA 1 “…*where I sit for now decisions are made mostly it is done at the Casco and the Dasco level. We only share forum on how they implement decisions made*… “ ISL 3. Community participation was also minimum, this was evident by communication approach used by facility to engage the community. A scenario described by a key informant: “…*community involvement is through sensitization of CHWs. If there is anything that we want to share, the CHVs inform the community*…" MSA 4.

### Leadership commitment and actions

from interviews conducted with key informants, the findings suggest that political commitment and regular revision of key policy documents has influenced stigma reduction and ownership in the implementation of HIV/AIDS integration policy, some of the respondents said: “*in terms of integration the leadership has tried to see how using the same policy documents has improved services..uuh and we see how different actors state and non-state actors contributing in terms of treatment and care….*" ICC 1 “…*we have seen where the first lady has taken launch of the Beyond Zero which is showing …mmh political commitment and support also there has been the revision of the key policy documents and in terms of service delivery where everybody …borrowing from the constitution irrespective of …without any judgment everybody is eligible to highest quality in terms of standards to a medical care, and we should not be having or stigmatizing…Then we have also seen in terms of leadership, where at various departments, we have focal people or focal persons that should be providing…Uhh or taking ownership of the implementation of the services.* ” LKN 8.

### Implementation status of integration framework

a number of health workers and key informants interviewed agree that integration has brought about changes reflected as effectiveness and efficiency. However, some facilities and county consolidated information indicate challenges in reaching expected standards in delivering integrated services as described here by respondents: “…*Service integration is like to offer all the services in one room, so as to reduce the time for the patient and the other thing I think sometime cost for the patient. Because to offer that service in one room. It becomes cost effective and efficient to the patient*. “ MSA 3. '''*there are some challenges that have risen in terms of the personnel. In terms of the service providers, there is shortage of drugs so the needs of the clients are not fully met… “* ISL 1. “…*patients are able to get services at one point, but maybe because of issues around infrastructure and staffing some are forced maybe to walk from MCH maybe to the lab or pharmacy. So just because of infrastructure and issues around staffing, we can´t or maybe we do not offer enough.”* LTK 1.

## Discussion

Health centres and hospitals both at sub-county and county level had a consistent response in existence of organizational structure on a positive note. This is an indicator of maturity in health system strengthening, attributable to efforts in devolution and implementation of integration frameworks. The two approaches confer autonomy to different level of healthcare institutions to perform specific functions which translates to some level of ownership and automatically invoking implementation of management structures [1,16]. Clarity of organization structure were at different level across levels of care, health centres experienced lesser appreciation and an upward trend observed towards hospital at sub-county and county level. Hospitals, have more functional roles, bigger resource capacity and a larger population accessing healthcare services hence there is deliberate effort to have a more differentiated and clear structure for efficient operations [17-19]. Ease of work with the existing structure was more evident at lower levels of care compared to hospitals at sub county and county level.

In concurrence with existing literature, larger organization with more functional roles have an increase in hierarchical structure, which in most cases are cumbersome and may be non-responsive to dynamics that are common in health systems hence the complexity in work environment [20]. Overall, there study provides evidence that organization structure at all levels of care, play a key role in providing directions and contextual interaction with the health system that is important in access of healthcare services [21]. Majority (80%) of the healthcare institution made deliberate effort to contact and make follow up with partners in the collaborative circles. Partnership is seen to have created synergy in the implementation of integration framework, particularly in Human Resources for Health (HRH) and Health Information System (HIS). Hospitals at county and referral level had more collaborative arrangement compared to health centres. Similarly, the capital Nairobi and Homabay had more collaborative arrangements. This can be directly linked to high resources needs given the population numbers and prevalence [22-26].

Collaboration had a highly coordinated multisectoral dimension and was mainly between health institution, particularly when making referrals and involvement of donor agencies in the various aspects of treatment and care. Level of participation in decision making was averagely high at 56% and the remaining, near half either reported medium level of participation or neutral. The perception on non-representation in decision making was drawn to the fact that most resources needs were not met as per the request and their opinion was not reflected in the final decision. More so, the community engagement was done through Community Health Volunteers (CHVs). Lack of total involvement of HW, community and patient undermine the principle of sustainable improvement in the delivery quality care to population in need [27].

Commitment and political influence proved to be key leadership roles in reduction of stigma and successful implementation of global health policies geared toward improved access of health care services [28-30]. This study draws evidence of in-depth engagement of leadership in political and technical action to improve access healthcare services particularly PMTCT. The commitment goes further to ensure implementation of context specific policy frameworks which is likely to be reflected in quality care and improved maternal health outcome. Integration framework intend to deliver services in consolidated approach for purposes of effectiveness and efficiency, which has been achieved to some extent. However, implementation status of integration varied across different levels of care and counties. Similarly so, the leadership action and strategies in implementation of policy framework was varied.

### Study limitation

the research findings was dependent on having access to people, organizations and documents. Generally, policy analysis has been known over time to be a sensitive area and this in most circumstances led to information insufficiency as data and retrieval of program/policy documents are restricted. The researcher was alive to the fact that individuals and institutional were keen to protect unknown interests. In this study, 16 key informant interviews were conducted with hospital/facility staff and donor agencies who provided in-depth information on HIV/AIDS services to fill the gaps arising from administering questionnaire and programme reports reviewed. To ensure acceptability and ownership of study findings, the research targeted multiple stakeholders to be among the pool of respondents and the study variables focused only health systems issues, while ensuring neutral position in matters related to individual leadership and politics.

## Conclusion

Clear and functional organization structure exists in all levels of healthcare institutions with ease of work diminishing towards higher levels of care, considered to have high workload and specialized care and these are a reflection of complexities in hierarchical structures particularly in higher levels of care. Deliberate efforts to realize coordinated and harmonious collaboration resulted to synergy in health information and health workforce management. Level of participation in decision making was likely to be reflected in implementation plans and it created perceptions in ownership and acceptance of strategies for achievement of health goals. Similarly, political commitment had greater influence in policy processes and stigma reduction that facilitate successful implementation of integration framework.

### Key messages

decentralization in complex hierarchical structure is necessary for smooth operations needed for delivery of quality care; collaboration and stakeholder involvement in decision making process, creates synergy in resources capacity and creates ownership in the implementation of healthcare goals; leaders need to commit by taking active political and technical role in the design and operationalization health systems framework; policy makers need to seek for up to date information on population´s demographic and disease characteristics to contextualized implementation of policy framework and realization of sustainable healthcare goals.

### What is known about this topic

- Studies have provided evidence that implementation of services integration policy framework are varied and the variations were associated with limitation in resources;
- Political stability and environmental factors may hinder or promote implementation of health policy.

### What this study adds

- This study provides comprehensive description of the systemic issues in system structures and processes, which include collaboration and decision-making in healthcare organizations and brings an understanding of health systems governance role in HIV/AIDS Services integration framework;
- This study provides understanding of good governance and actions required in policy processes necessary to bring about desired policy outcomes and achievement of health goals.

## Competing interests

The author declares no competing interests.
